# Topological phase transitions and chiral inelastic transport induced by the squeezing of light

**DOI:** 10.1038/ncomms10779

**Published:** 2016-03-02

**Authors:** Vittorio Peano, Martin Houde, Christian Brendel, Florian Marquardt, Aashish A. Clerk

**Affiliations:** 1 Institute for Theoretical Physics, University of Erlangen-Nürnberg, Staudtstr. 7, 91058 Erlangen, Germany; 2 Department of Physics, McGill University, 3600 rue University, Montreal, Quebec, Canada H3A 2T8; 3 Max Planck Institute for the Science of Light, Günther-Scharowsky-Straße 1/Bau 24, 91058 Erlangen, Germany

## Abstract

There is enormous interest in engineering topological photonic systems. Despite intense activity, most works on topological photonic states (and more generally bosonic states) amount in the end to replicating a well-known fermionic single-particle Hamiltonian. Here we show how the squeezing of light can lead to the formation of qualitatively new kinds of topological states. Such states are characterized by non-trivial Chern numbers, and exhibit protected edge modes, which give rise to chiral elastic and inelastic photon transport. These topological bosonic states are not equivalent to their fermionic (topological superconductor) counterparts and, in addition, cannot be mapped by a local transformation onto topological states found in particle-conserving models. They thus represent a new type of topological system. We study this physics in detail in the case of a kagome lattice model, and discuss possible realizations using nonlinear photonic crystals or superconducting circuits.

## Abstract (web summary)

Most known topological states of light are in the end closely analogous to fermionic states. Here, the authors show that the squeezing of light can lead to the formation of photonic topological states which do not have any fermionic counterpart yet support unusual chiral edge states.

## Graphical abstract

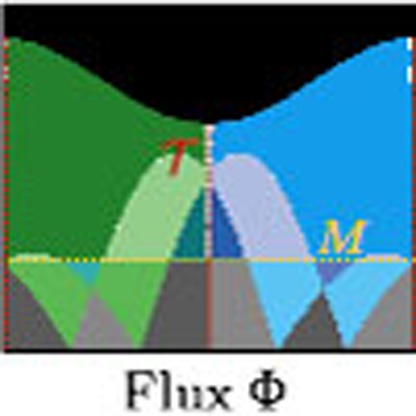

Waves are not only ubiquitous in physics, but the behaviour of linear waves is also known to be very generic, with many features that are independent of the specific physical realization. This has traditionally allowed us to transfer insights gained in one system (for example, sound waves) to other systems (for example, matter waves). That strategy has even been successful for more advanced concepts in the field of wave transport. One important recent example of this kind is the physics of topological wave transport, where waves can propagate along the boundaries of a sample, in a one-way chiral manner that is robust against disorder scattering. While first discovered for electron waves, this phenomenon has by now also been explored for a variety of other waves in a diverse set of systems, including cold atoms^1^, photonic systems^2^ and more recently phononic systems^3^,^4^,^5^,^6^,^7^,^8^,^9^.

In the case of topological wave transport, the connection between waves in different physical implementations can actually be so close that the calculations turn out to be the same. In particular, if we are dealing with matter waves moving in a periodic potential, the results do not depend on whether they are bosons or fermions, as long as interactions do not matter. The single-particle wave equation to be solved happens to be exactly the same. This has allowed to envision and realize photonic analogues of quantum-Hall effect^10^,^11^,^12^,^13^,^14^,^15^,^16^,^17^,^18^, the spin Hall effect^19^,^20^,^21^,^22^, Floquet topological insulators^23^,^24^ and even Majorana-like modes^25^. More generally, the well-known classification of electronic band structures based on the dimensionality and certain generalized symmetries^26^ directly applies to photonic systems provided that the particle number is conserved. As we now discuss, this simple correspondence will fail in the presence of squeezing.

Consider the most general quadratic Hamiltonian describing photons in a periodic potential in the presence of parametric driving:


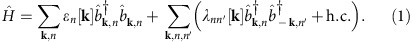


The first term describes a non-interacting photonic band structure, where 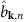 annihilates a photon with quasimomentum **k** in the *n*-th band. The remaining two-mode squeezing terms are induced by parametric driving and do not conserve the excitation number. As we discuss below, they can be controllably realized in a number of different photonic settings. While superficially similar to pairing terms in a superconductor, these two-mode squeezing terms have a profoundly different effect in a bosonic system, as there is no limit to the occupancy of a particular single-particle state. They can give rise to highly entangled ground states, and even to instabilities.

Given these differences, it is natural to ask how anomalous pairing terms can directly lead to topological phases of light. In this work, we study the topological properties of two-dimensional photonic systems described by Equation (1), in the case where the underlying particle-conserving band structure has no topological structure, and where the parametric driving terms do not make the system unstable. We show that the introduction of particle non-conserving terms can break time-reversal symmetry (TRS) in a manner that is distinct from having introduced a synthetic gauge field, and can lead to the formation of bands having a non-trivial pattern of (suitably defined) quantized Chern numbers. This in turn leads to the formation of protected chiral edge modes: unlike the particle-conserving case, these modes can mediate a protected inelastic (but still coherent) scattering mechanism along the edge (that is, a probe field injected into the edge of the sample will travel along the edge, but emerge at a different frequency). In general, the topological phases we find here are distinct both from those obtained in the particle-conserving case, and from those found in topological superconductors. We also discuss possible realizations of this model using a nonlinear photonic crystal or superconducting microwave circuits. Finally, we discuss the formal analogies and crucial differences between the topological phases of light investigated here and those recently proposed for other kinds of Bogoliubov quasiparicles^27^,^28^,^29^,^30^,^31^ (see Discussion section).

## Results

### Kagome lattice model

**Figure 1:**
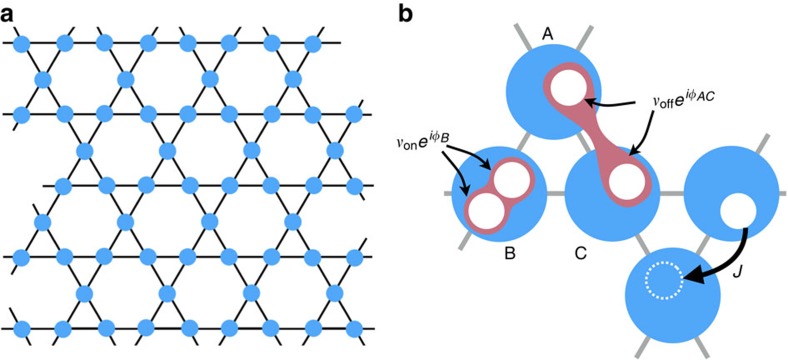
Setup figure. (**a**) An array of nonlinear cavities forming a kagome lattice. (**b**) Photons hop between nearest-neighbour sites with rate *J*. Each cavity is driven parametrically leading to the creation of photon pairs on the same lattice site (rate *ν*_on_) and on nearest-neighbour sites (rate *ν*_off_). A spatial pattern of the driving phase is imprinted on the parametric interactions, breaking the time-reversal symmetry (but preserving the 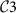 rotational symmetry).

For concreteness, we start with a system of bosons on a kagome lattice (Fig.1),


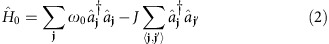


(we set *ℏ*=1). Here we denote by 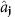 the photon annihilation operator associated with lattice site **j**, where the vector site index has the form **j**=(*j*_1_,*j*_2_,*s*). *j*_1_,*j*_2_∈*Z* labels a particular unit cell of the lattice, while the index *s*=*A*,*B*,*C* labels the element of the sublattice. 〈**j,j′**〉 indicates the sum over nearest neighbours, and *J* is the (real valued) nearest-neighbour hopping rate; *ω*_0_ plays the role of an onsite energy. As there are no phases associated with the hopping terms, this Hamiltonian is time-reversal symmetric and topologically trivial. We chose the kagome lattice because it is directly realizable both in quantum optomechanics^5^ and in arrays of superconducting cavity arrays^13^,^16^; it is also the simplest model where purely local parametric driving can result in a topological phase.

We next introduce quadratic squeezing terms to this Hamiltonian that preserve the translational symmetry of the lattice and that are no more non-local than our original, nearest-neighbour hopping Hamiltonian:


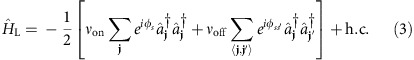


Such terms generically arise from having a nonlinear interaction with a driven auxiliary pump mode (which can be treated classically) on each site, see for example, ref. 32. As we discuss below, the variation in phases in 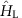 from site to site could be achieved by a corresponding variation of the driving phase of the pump. Note that we are working in a rotating frame where this interaction is time independent, and thus *ω*_0_ should be interpreted as the detuning between the parametric driving and the true onsite (cavity) frequency *ω*_cav_ (that is, *ω*_0_=*ω*_cav_−*ω*_L_/2, where the parametric driving is at a frequency *ω*_L_). The parametric driving can cause the system to become unstable; we will thus require that the onsite energy (that is, parametric drive detuning) *ω*_0_ be sufficiently large that each parametric driving term is non-resonant enough to ensure stability. If one keeps *ω*_0_ fixed, this means that the parametric driving amplitudes *ν*_on_, *ν*_off_ will be limited to some fraction of *ω*_0_ (the particular value of which depends on *J*, Supplementary Note 1).

For a generic choice of phases in the parametric driving Hamiltonian of Equation (3), it is no longer possible to find a gauge where 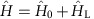 is purely real when expressed in terms of real-space annihilation operators: hence, even though the hopping Hamiltonian 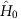 corresponds to strictly zero flux, the parametric driving can itself break TRS. In what follows, we will focus for simplicity on situations where time reversal and particle conservation are the only symmetries broken by the parametric driving: they will maintain the inversion and 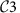 rotational symmetry of the kagome lattice. We will also make a global gauge transformation so that *ν*_off_ is purely real, while *ν*_on_=|*ν*_on_|*e*^*iϕ*^_*ν*_. In this case, the only possible choices for the *φ* phases have the form (*φ*_*A*_,*φ*_*B*_,*φ*_*C*_)=(*φ*_*AB*_,*φ*_*BC*_,*φ*_*CA*_)=±(0,*δ*,2*δ*) with *δ*=2*πm*_*ν*_/3, where *m*_*ν*_ is an integer and is the vorticity of the parametric driving phases. We stress that these phases (and hence the sign of the TRS breaking) are determined by the phases of the pump modes used to generate the parametric interaction.

### Gap opening and non-trivial topology


**Figure 2:**
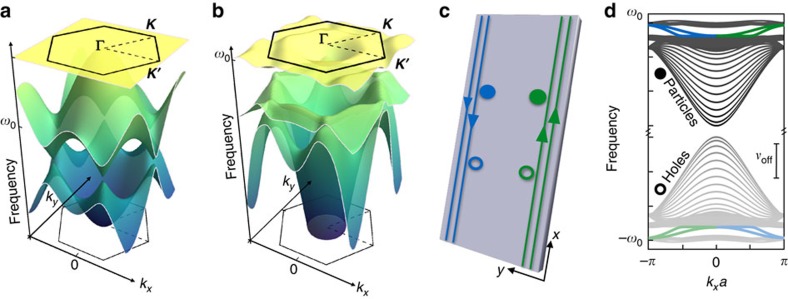
Topological Band structure. (**a**,**b**) 3D plots of the bulk band structure. The hexagonal Brillouin zone is also shown. (**a**) In the absence of parametric driving, neighbouring bands touch at the rotational symmetry points ***K***, ***K***′ and **Γ**. (**b**) The parametric driving opens a gap between subsequent bands. For the chosen parameters, there is a global band gap between the second and third band. (**d**) Hole and particle bands, ±*E*_*m*_[*k*_*x*_], in a strip geometry (sketched in **c**). The line intensity is proportional to the weight of the corresponding resonance in the photon spectral function, Supplementary Note 1. The edge states localized on the right (left) edge, plotted in green (blue), have positive (negative) velocity. Parameters: Hopping rate *J*=0.02*ω*_0_ (*ω*_0_ is the onsite frequency); (**b**,**d**), the parametric couplings are *ν*_on_=−0.085*ω*_0_ and *ν*_off_=0.22*ω*_0_.

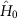
 is the standard tight-binding kagome Hamiltonian for zero magnetic field, and does not have band gaps: the upper and middle bands touch at the symmetry point **Γ**≡(0,0), whereas the middle and lower bands touch at the symmetry points ***K***=(2*π*/3,0) and ***K*****′**=(*π*/3,*π*/(3)^1/2^) where they form Dirac cones (Fig. 2a).

Turning on the pairing terms, the Hamiltonian 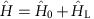 can be diagonalized in the standard manner as 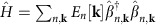, where the 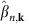 are canonical bosonic annihilation operators determined by a Bogoliubov transformation of the form (see Methods section):


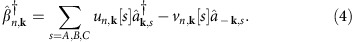


Here 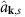 are the annihilation operators in quasimomentum space, and *n*=1,2,3 is a band index; we count the bands by increasing energy. The photonic single-particle spectral function now shows resonances at both positive and negative frequencies, ±*E*_*n*_[*k*], corresponding to particle- and hole-type bands, Fig. 2d. Because of the TRS breaking induced by the squeezing terms, the band structure described by *E*_*n*_[**k**] now exhibits gaps, Fig. 2b; furthermore, for a finite sized system, one also finds edge modes in the gap, Fig. 2d.

The above behaviour suggests that the parametric terms have induced a non-trivial topological structure in the wavefunctions of the band eigenstates. To quantify this, we first need to properly identify the Berry phase associated with a bosonic band eigenstate in the presence of particle non-conserving terms. For each **k**, the Bloch Hamiltonian 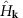 corresponds to the Hamiltonian of a multi-mode parametric amplifier. Unlike the particle-conserving case, the ground state of such a Hamiltonian is a multi-mode squeezed state with non-zero photon number; it can thus have a non-trivial Berry's phase associated with it when **k** is varied, Supplementary Note 2. The Berry phase of interest for us will be the difference of this ground state Berry phase and that associated with a single quasiparticle excitation. One finds that the resulting Berry connection takes the form


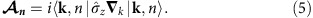


Here the six vector of Bogoliubov coefficients |**k**, *n*〉≡(*u*_*n*,**k**_[*A*], *u*_*n*,**k**_[*B*], *u*_*n*,**k**_[*C*], *v*_*n*,**k**_[*A*], *v*_*n*,**k**_[*B*], *v*_*n*,**k**_[*C*]) plays the role of a singe-particle wavefunction, and 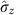 acts in the particle-hole space, associating +1 to the *u* components and −1 to the *v* components, see Methods section. These effective wavefunctions obey the symplectic normalization condition


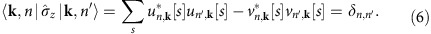


Having identified the appropriate Berry connection for a band eigenstate, the Chern number for a band *n* is then defined in the usual manner:


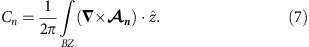


The definition in Eq. (5) agrees with that presented in ref. 27 and (in one-dimension) ref. 29; standard arguments^27^ show that the *C*_*n*_ are integers with the usual properties. We note that, as for superconductors, breaking the *U*(1) (particle-conservation) symmetry remains compatible with a first-quantized picture after doubling the number of bands. The additional hole bands are connected to the standard particle bands by a particle–hole symmetry; see Methods section. In bosonic systems, the requirement of stability generally implies that particle and hole bands can not touch; this is true for our system. Thus, the sum of the Chern numbers over the particle bands (with *E*>0) must be zero, and there cannot be any edge states with energies below the lowest particle bulk band (or in particular, at zero energy); Supplementary Note 1.

In the special case where we only have onsite parametric driving (that is, *ν*_off_=0,*ν*_on_≠0), the Chern numbers can be calculated analytically (Supplementary Note 3). They are uniquely fixed by the pump vorticity. If *m*_*ν*_=0, we have TRS and the band structure is gapless, while for *m*_*ν*_=±1, ***C***=(∓1, 0, ±1). This set of topological phases also occurs in a particle-number conserving model on the kagome lattice with a staggered magnetic field, that is, the Oghushi–Murakami–Nagaosa (OMN) model of the anomalous quantum-Hall effect^33^,^34^.

**Figure 3:**
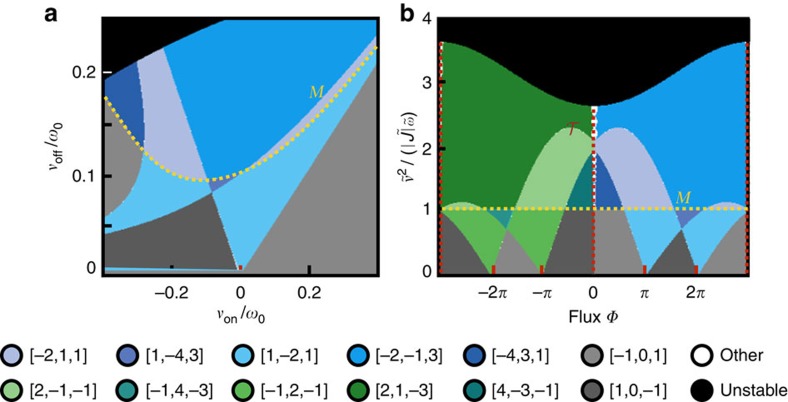
Symplectic Topological phase diagrams. (**a**)Topological phase diagram for the parametrically driven kagome lattice model. The *y* (*x*) axis corresponds to the strength of the onsite parametric drive *ν*_on_ (offsite parametric drive *ν*_off_), and different colours correspond to different triplets ***C***=(*C*_1_,*C*_2_,*C*_3_) of Chern numbers for the three bands of the model. Note that only the grey and dark-grey phases are found in the particle-conserving version of our model with a staggered field. We have fixed the hopping rate *J*/*ω*_0_=0.02, and the vorticity of the pump *m*_*ν*_=1. (**b**) Same phase diagram, but now plotted in terms of the effective flux 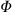 and effective parametric drive 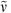 experienced by *α* quasiparticles.

In the general case, where we include offsite parametric driving, entirely new phases appear. We have computed the Chern numbers here numerically, using the approach of ref. 35. In Fig. 3a, we show the topological phase diagram of our system, where *J*/*ω*_0_ and *m*_*ν*_ are held fixed, while the parametric drive strengths *ν*_on_,*ν*_off_ are varied. Different colours correspond to different triplets ***C***≡(*C*_1_,*C*_2_,*C*_3_) of the band Chern numbers, with grey and dark-grey corresponding to the two phases already present in the OMN model. Strikingly, a finite off-diagonal coupling *ν*_off_ generates a large variety of phases which are not present in the OMN model, including phases having bands with |*C*_*n*_|>1. The border between different topological phases represent topological phase transitions, and correspond to parameter values where a pair of bands touch at a particular symmetry point; we discuss this further below. Via a standard bulk-boundary correspondence (Supplementary Note 4), the band Chern numbers for a particular phase determine the number of protected edge states that will be present in a system with a boundary; as usual, the number of edge states in a particular bandgap is obtained by summing the Chern numbers of lower-lying bands. We discuss these edge states in greater detail in a following subsection. Finally, the black regions in the phase diagram indicate regimes of instability, which occur when the parametric driving strength becomes too strong.

### Dressed-state picture

To gain further insight into the structure of the topological phases found above, it is useful to work in a dressed-state basis that eliminates the local parametric driving terms from our Hamiltonian. We thus first diagonalize the purely local terms in the Hamiltonian; for each lattice site **j** we have


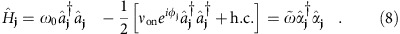


Here 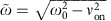, and the annihilation operators 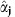 are given by a local Bogoliubov (squeezing) transformation 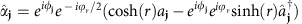, where the squeezing factor *r* is


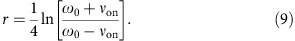


On a physical level, the local parametric driving terms attempt to drive each site into a squeezed vacuum state with squeeze parameter *r*; the 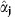 quasiparticles correspond to excitations above this reference state. Note that we have included an overall phase factor in the definition of the 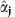, which will simplify the final form of the full Hamiltonian.

In this new basis of local quasiparticles, our full Hamiltonian takes the form


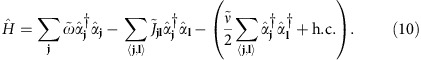


The transformation has mixed the hopping terms with the non-local parametric terms: The effective counter-clockwise hopping matrix element is


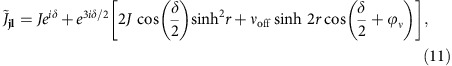


and the magnitude of the effective non-local parametric driving is


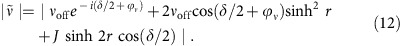


Note that the phase of 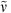 can be eliminated by a global gauge transformation, and hence it plays no role; we thus take 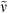 to be real in what follows.

Our model takes on a much simpler form in the new basis: the onsite parametric driving is gone, and the non-local parametric driving is real. Most crucially, the effective hoppings can now have spatially varying phases, which depend both on the vorticity of the parametric driving in 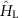 (through *δ*), and the magnitude of the onsite squeezing (through *r*). In this transformed basis, the effective hopping phases are the only route to breaking TRS. Our model has thus been mapped onto the standard OMN model for the anomalous quantum-Hall effect, with an additional (purely real) nearest-neighbour two-mode squeezing interaction. In the regime where the parametric interactions between the 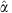 quasiparticles are negligible (Supplementary Note 3), the complex phases correspond in the usual manner to a synthetic gauge field (that is, the effective flux *Φ* piercing a triangular plaquette would be *Φ*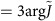). In other words, the squeezing creates a synthetic gauge field for Bogoliubov quasiparticles. However, in the presence of substantial parametric interaction between 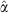 quasiparticles, the parameter *Φ* can not be interpreted anymore as a flux: a flux of 2*π* can not be eliminated by a gauge transformation because the complex phases reappear in the parametric terms. In that case, only a periodicity of 6*π* in *Φ* is retained, since that corresponds to having trivial hopping phases of 2*π*.

Understanding the topological structure of this transformed Hamiltonian is completely sufficient for our purposes: one can easily show that the Chern number of a band is invariant under any local Bogoliubov transformation, hence the Chern numbers obtained from the transformed Hamiltonian in Equation (8) will coincide exactly with those obtained from the original Hamiltonian in Equation (3). We thus see that the topological structure of our system is controlled completely by only three dimensionless parameters: the flux *Φ* (associated with the hopping phases), the ratio 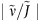, and the ratio 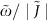.

The topological phase diagram for the effective model is shown in Fig. 3b. Again, one sees that as soon as the effective non-local parametric drive 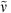 is non-zero, topological phases distinct from the standard (particle-conserving) OMN model are possible. The sign of the parametric pump vorticity *m*_*ν*_ determines the sign of the effective flux *Φ*, c.f. Equation (11). As such, the right half of Fig. 3b (corresponding to *Φ* > 0) is a deformed version of the phase diagram of the original model for pump vorticity *m*_*ν*_=1, as plotted in Fig. 3a. Changing the sign of *m*_*ν*_ (and hence *Φ*) simply flips the sign of all Chern numbers, Supplementary Note 3.

Our effective model provides a more direct means for understanding the boundaries between different topological phases. Most of these are associated with the crossing of bands at one or more high-symmetry points in the Brillouin zone; this allows an analytic calculation of the phase boundary (Supplementary Note 3). Perhaps most striking in Fig. 3b is the horizontal boundary (labelled 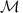), occurring at a finite value of the effective offsite parametric drive, 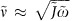. This boundary is set by the closing of a band gap at the ***M*** points; as these points are associated with the decoupling of one sublattice from the other two, this boundary is insensitive to the flux *Φ*. Similarly, the vertical line labelled 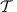 denotes a line where the system has TRS, and all bands cross at the symmetry points ***K***, ***K***′ and **Γ**. The case of zero pump vorticity *m*_*ν*_=0 (not shown) is also interesting. Here the effective flux *Φ* depends on the strength of the parametric drivings, but is always constrained to be 0 or 3*π*. This implies that the effective Hamiltonian has TRS, even though the original Hamiltonian may not (that is, if Im ν_off_≠0, the original Hamiltonian does not have TRS). For *m*_*ν*_=0, the parametric drivings do not open any band gap and the Chern numbers are not well defined.

### Edge states and transport

Despite their modified definition, the Chern numbers associated with our Bogoliubov bands still guarantee the existence of protected chiral edge modes in a system with boundaries via a standard bulk-boundary correspondence, see Supplementary Note 4. These states can be used to transport photons by exciting them with an auxiliary probe laser beam, which is focused on an edge site and at the correct frequency. The lack of particle-number conservation manifests itself directly in the properties of the edge states: along with the standard elastic transmission they can also mediate inelastic scattering processes. In terms of the original lab frame, light injected at a frequency *ω*_p_ can emerge on the edge at frequency *ω*_L_−*ω*_p_, where *ω*_L_ is the frequency of the laser parametrically driving the system. This is analogous to the idler output of a parametric amplifier. Here both signal and idler have a topologically protected chirality.

**Figure 4:**
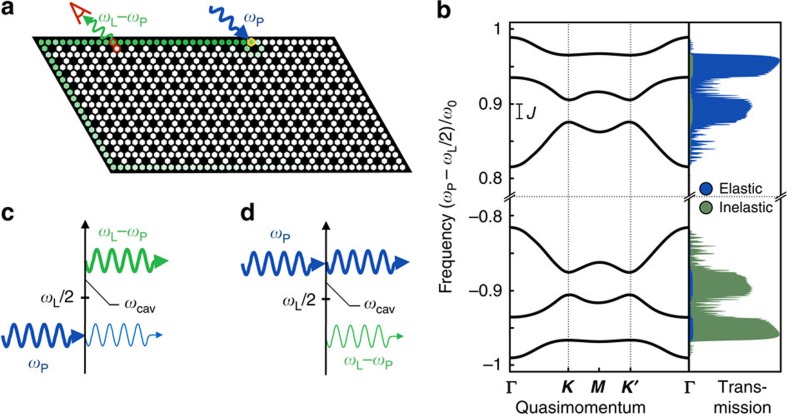
Topologically protected transport in a finite system. (**a**) A probe beam at frequency *ω*_p_ inside the bulk band gap is focused on a site (marked in yellow) at the edge of a finite sample. The probability map of the light transmitted inelastically at frequency *ω*_L_−*ω*_p_ (where *ω*_L_ is the frequency of the drive tone applied to the auxiliary pump modes) clearly shows that the transport is chiral. (**b**) The elastic and inelastic transmission probability to a pair of sites along the edges (indicated in red in **a**) is plotted in blue and green, respectively. A cut through the bulk bands is shown to the left. (**c**,**d**) Sketch of the relevant scattering processes and energy scales. The inelastic (elastic) transmission has a larger rate when the light is injected in the hole (particle) band gap. Parameters: Hopping rate *J*=0.02*ω*_0_ (*ω*_0_ is the onsite frequency), parametric couplings *ν*_on_=0.4*ω*_0_ and *ν*_off_=0.02*ω*_0_, optical decay rate *κ*=0.001*ω*_0_. (**a**) *ω*_p_−*ω*_L_/2=0.95*ω*_0_.

Shown in Fig. 4 are the results of a linear response calculation describing such an experiment, applied to a finite system with corners. We incorporate a finite photon decay rate *κ* in the standard input–output formalism, see Methods section. Narrow-band probe light inside a topological band gap is applied to a site on the edge, and the resulting inelastic transmission probabilities to each site on the lattice are plotted, Fig. 4a. One clearly sees that the probe light is transmitted in a unidirectional way along the edge of the sample, and is even able to turn the corner without significant backscatter. The corresponding elastic transmission (not shown) is also chiral and shows the same spatial dependence. In Fig. 4b we show the elastic and inelastic transmissions to the sites indicated in red (rescaled by the overall transmission, 1−*R* where *R* is the reflection probability at the injection site) as a function of the probe frequency *ω*_p_. By scanning the laser probe frequency, one can separately address particle and hole band gaps. The relative intensity of the inelastic scattering component is highly enhanced when the probe beam is inside a hole band gap, see also the sketches in Fig. 4c,d. When the parametric interaction between the 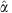 quasiparticles is negligible, the ratio of elastic and inelastic transmissions depends only on the squeezing factor *r*, (c.f. Equation (9)), see Methods section.

### Physical realization

Systems of this type could be implemented in photonic crystal coupled cavity arrays^36^ fabricated from nonlinear optical *χ*^2^ materials^37^,^38^,^39^. The array of optical modes participating in the transport would be supplemented by pump modes (resonant with the pump laser at twice the frequency). One type of pump mode could be engineered to be spatially co-localized with the transport modes (*ν*_on_ processes), while others could be located in-between (*ν*_off_). The required periodic phase pattern of the pump laser can be implemented using spatial light modulators or a suitable superposition of several laser beams impinging on the plane of the crystal. One method for realizing the required kagome lattice of defect cavities was discussed in ref. 5. Optomechanical systems offer another route towards generating optical squeezing terms^40^,^41^, via the mechanically induced Kerr interaction, and this could be exploited to create an optomechanical array with a photon Hamiltonian of the type discussed here. Alternatively, these systems can be driven by two laser beams to create phononic squeezing terms^42^. A fourth alternative consists in superconducting microwave circuits of coupled resonators, where Josephson junctions can be embedded to introduce *χ*^2^ and higher order nonlinearities, as demonstrated in refs 43, 44. Kagome lattices of superconducting resonators have recently been implemented^45^.

## Discussion

Before concluding, it is worthwhile to discuss the connections between our work and other recent studies. A Hamiltonian of the general form of Equation (1) arises naturally in the mean-field description of a Bose-condensed phase. In this setting, the anomalous pairing terms describe the interactions with the condensate treated at the mean-field level. A few recent studies have proposed to take advantage of these interactions to selectively populate topological edge states^28^,^30^ or, closer to our study, to induce novel topological phases. These include a study of a magnonic crystal^27^, as well as general Bose–Einstein condensates in one-dimension^29^ and in two-dimensions^31^.

There are some crucial differences between the above studies and our work. In our case, Equation (1) describes the real particles of our system, not quasiparticles defined above some background. This difference is not just a question of semantics: in our case, topological effects can directly be seen by detecting photons, whereas in refs 29, 31, one would need to isolate the contribution of a small number of Bogoliubov quasiparticles sitting on a much larger background of condensed particles. In addition, in our work the pairing terms in Equation (1) are achieved by driving the system, implying that negative and positive frequencies are clearly physically distinguished (that is, they are defined relative to a non-zero pump frequency). This is at the heart of the topologically protected inelastic scattering mechanism we describe, and is something that is not present in previous studies.

Our work opens the door to a number of interesting new directions. On the more practical side, one could attempt to exploit the unique edge states in our system to facilitate directional, quantum-limited amplification. On the more fundamental level, one could use insights from the corresponding disorder problem^46^ and attempt to develop a full characterization of particle non-conserving bosonic topological states that are described by quadratic Hamiltonians. This would then be a counterpart to the classification already developed for fermionic systems^26^.

## Methods

### Bogoliubov transformation and first-quantized picture

We find the normal mode decompositions leading to the band structures in Fig. 2 and the topological phase diagrams in Fig. 3 by introducing a first-quantized picture. Since the relevant Hamiltonians do not conserve the excitation number, this is only possible after doubling the degrees of freedom. This is achieved by grouping all annihilation operators with quasimomentum **k** and the creation operators with quasimomentum −**k** in the 2*N* vector of operators 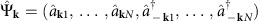 (where *N* is the unit cell dimension), and by casting the second quantized Hamiltonian 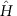 in the form


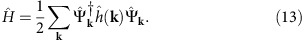


The 2*N* × 2*N* hermitian matrix 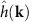 plays the role of a single-particle Hamiltonian and is referred to as the Bogoliubov de Gennes Hamiltonian. By definition of the normal modes 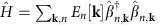, we have 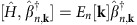. By plugging into the above equation the Bogoliubov ansatz Equation (4) one immediately finds


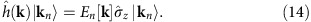


Likewise, from 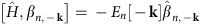 one finds


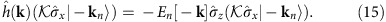


Here denotes the complex conjugation and the matrix 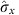 exchanges the *u*'s and the *v*'s Bogoliubov coefficients,


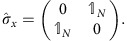


Thus, the spectrum of the 2*N* matrix 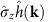 is formed by the set of 2*N* eigenenergies *E*_*n*_[**k**] (belonging to the particle bands) and −*E*_*n*_[−**k**] (belonging to the hole bands). *Vice versa*, to calculate the eigenenergies *E*_*n*_[**k**] and −*E*_*n*_[−**k**] and the vector of Bogoliubov coefficients in Equation (4), we have to solve the eigenvalue problem


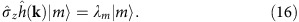


The solutions we are interested in should also display the symplectic orthonormality relations Equation (6).

We note in passing that so far we have implicitly assumed that the normal mode decomposition is possible. However, this is not always the case. When the matrix 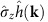 has any complex eigenvalue, the Hamiltonian is unstable. Moreover, at the border of the stable and unstable parameter regions, the matrix 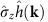 is not diagonalizable. The Supplementary Note 1 contains a stability analysis of our specific model.

In the stable regime of interest here the matrix 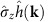 is diagonalizable and all its eigenvalues are real. In this case, its eigenvectors |*m*〉 can be chosen to be mutually 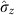 orthogonal. In addition, there are exactly *N* positive (negative) norm eigenvectors. Thus, it is always possible to enforce the symplectic orthonormality relations Equation (6) by identifying the (appropriately normalized) positive and negative norm solutions with |**k**_*n*_〉 and 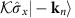, respectively. The corresponding eigenvalues are then to be identified with *E*_*n*_[**k**] (particle band structure) and −*E*_*n*_[−**k**] (hole band structure), respectively

*Particle–hole symmetry*. The Bogoliubov de Gennes Hamiltonian has the generalized symmetry 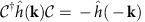 where the charge conjugation operator 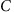 is anti-unitary and *C*^2^=_2*N*_. Thus, our system represents the bosonic analogue of a superconductor in the Class *D* of the standard topological classification. This is a simple consequence of the doubling of the degrees of freedom in the single-particle picture. It simply reflects that the set of ladder operators 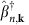 and 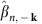 calculated from 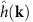 are the adjoint of the set of operators 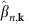 and 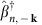 calculated from 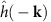.

### Details of the transport calculations

In our transport calculations we have included photon decay. We adopt the standard description of the dissipative dynamics of photonic systems in terms of the Langevin equation and the input–output theory^47^, for each site:


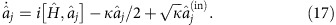


In practice, we consider an array of detuned parametric amplifiers with intensity decay rate *κ* and add to the standard description of each parametric amplifier the inter-cell coherent coupling described in the main text. The last term describes the influence of the input field 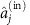 injected by an additional probe drive including also the environment vacuum fluctuations. The field 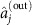 leaking out of each cavity at site *j* is given by the input–output relations


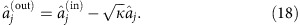


The above formulas give an accurate description of a photonic system where the intrinsic losses during injection and inside the system are negligible. Intrinsic photon absorption can be incorporated by adding another decay channel to the equation for the light field. It reduces the propagation length but does not change qualitatively the dynamics.

In Fig. 3, we show the probabilities *T*_E_(*ω*, *l*, *j*) and *T*_I_(*ω*, *l*, *j*) that a photon injected on site *j* with frequency *ω*_in_=*ω*+*ω*_L_/2 is transmitted elastically (at frequency *ω*+*ω*_L_/2) or inelastically (at frequency *ω*_L_/2−*ω*) to site *l* where it is detected. From the Kubo formula and the input–output relations we find


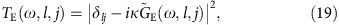



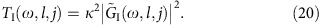


Here 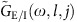 are the elastic and inelastic components of the Green's function in frequency space,


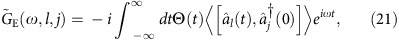



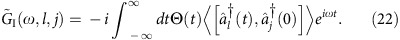


In a *N* site array with single-particle eigenstates |*n*〉=(*u*_*n*_[1],…,*u*_*n*_[*N*],*v*_*n*_[1],…,*v*_*n*_[*N*])^*T*^, the Green's functions read


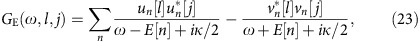



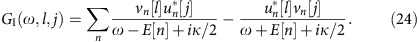


We note that for a probe field inside the bandwidth of the particle (hole) sector but far detuned from the hole (particle) sector, only the first (second) term of the summand in Equations (23) and (24) is resonant. Thus, as expected, the inelastic scattering is comparatively larger when the probe field is in the hole band gap.

It is easy to estimate quantitatively the relative intensities of elastically and inelastically transmitted light when the parametric interaction of the 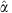 Bogoliubov quasiparticles is small (the regime where *Φ* can be interpreted as a synthetic gauge field experienced by the Bogoliubov quasiparticles). In this case, it is straightforward to show that |*v*_*n*_[*j*]/*u*_*n*_[*j*]|≈tanh*r* independent of the eigenstate *n* and the site *j*. By putting together Equations (20, 23, 24) and neglecting the off-resonant terms we find that for 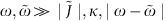,


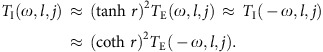


These analytical formulas agree quantitatively with the numerical results shown in Fig. 4b (note that in Fig. 4b the transmission at the output sites is rescaled by the overall transmission, ∑_*l* ≠ *j*_*T*_I_(*ω*, *l*, *j*)+*T*_E_(*ω*, *l*, *j*)).

## Additional information

**How to cite this article:** Peano, V. *et al*. Topological phase transitions and chiral inelastic transport induced by the squeezing of light. *Nat. Commun.* 7:10779 doi: 10.1038/ncomms10779 (2016).

## Supplementary Material

Supplementary Information Supplementary Notes 1-4 and Supplementary References
