# Supplementary material for: Topological phase transitions and chiral inelastic transport induced by the squeezing of light
Source: Nat Commun. 2016 Mar 2;7:10779. doi: 10.1038/ncomms10779 (PMC4778048; doi:10.1038/ncomms10779)
Supplement: Supplementary Information — Supplementary Notes 1-4 and Supplementary References [file ncomms10779-s1.pdf]

Supplementary Note 1: Details of the calculation of the band structure

Numerical calculation of the band structure

From Eqs. (2,3,13) of the main text, one can immediately derive the explicit expression of the Bogoliubov de Gennes Hamiltonian

$$\hat{h}(\mathbf{k}) = \begin{pmatrix} \omega_0 - J\hat{\tau}(\mathbf{k}) & h_L(\mathbf{k}) \\ h_L^\dagger(\mathbf{k}) & \omega_0 - J\hat{\tau}(\mathbf{k}) \end{pmatrix} \tag{1}$$

where $\hat{h}_L = -\nu_{\mathrm{on}}\hat{\Lambda}^{m_\nu} - \nu'_{\mathrm{off}}(\hat{\Lambda}^{m_\nu}\hat{\tau} + \hat{\tau}\hat{\Lambda}^{m_\nu})$ (m_ν is the pump vorticity). The matrices $\hat{\tau}(\mathbf{k})$ and $\hat{\Lambda}$ are single-particle operators acting on the sub-lattice degrees of freedom. They have matrix elements: $\Lambda_{AA} = 1$, $\Lambda_{BB} = e^{i2\pi/3}$, $\Lambda_{CC} = e^{-2i\pi/3}$, $\tau_{BA} = \tau^*_{AB} = 1 + e^{i\mathbf{k}\cdot\boldsymbol{a}_1}$, $\tau_{CB} = \tau^*_{BC} = 1 + e^{i\mathbf{k}\cdot\boldsymbol{a}_2}$, $\tau_{AC} = \tau^*_{CA} = 1 + e^{i\mathbf{k}\cdot\boldsymbol{a}_3}$ where $\boldsymbol{a}_1 = (-1, -\sqrt{3})$, $\boldsymbol{a}_2 = (2, 0)$, and $\boldsymbol{a}_3 = (-1, \sqrt{3})$ are lattice vectors. All other matrix elements are zero. Notice that $\hat{\Lambda}$ raises the quasi-angular momentum by one unit: $\hat{\Lambda}|p, m\rangle = |p, m+1\rangle$ where $|p, m\rangle = (1, \exp[i2m\pi/3], \exp[-i2m\pi/3], 0, 0, 0)$ and likewise for the holes. Here, $m = 0$ is the vortex free eigenstate, $m = 1$ has a vortex, and $m = -1$ an anti-vortex (m is defined modulo 2). Thus, a hole with quasimomentum m is converted into a particle with quasi-angular momentum $m + m_\nu$. In other words, a pair of down-converted photons have quasi-angular momenta $-m$ and $m_\nu + m$, respectively. The additional quasimomentum m_ν is provided by the pump photons. In order to write the off-diagonal parametric interaction compactly in terms of the quasi-angular momentum raising operator $\hat{\Lambda}$ we have introduced the rescaled off-diagonal parametric coupling $\nu'_{\mathrm{off}} = e^{-i\delta/2}\nu_{\mathrm{off}}$ for pump vorticity $m_\nu = \pm 1$ and $\nu'_{\mathrm{off}} = \nu_{\mathrm{off}}/2$ for pump vorticity $m_\nu = 0$.

For the effective model Equation (8) of the main text the Bogoliubov de Gennes Hamiltonian reads

$$\hat{\tilde{h}}(\mathbf{k}) = \begin{pmatrix} \tilde{\omega} - \tilde{J}\hat{\tilde{\tau}}(\mathbf{k}, \Phi) & -\tilde{\nu}\hat{\tau}(\mathbf{k}) \\ -\tilde{\nu}\hat{\tau}(\mathbf{k}) & \tilde{\omega} - \tilde{J}\hat{\tilde{\tau}}(\mathbf{k}, -\Phi) \end{pmatrix}. \tag{2}$$

Here, we have introduced the hopping matrix $\hat{\tau}(\mathbf{k}, \Phi)$ in the presence of the synthetic magnetic field flux Φ. It has the following non zero matrix elements: $\tilde{\tau}_{BA} = \tilde{\tau}^*_{AB} = e^{i\Phi/3}(1 + e^{i\mathbf{k}\cdot\boldsymbol{a}_1})$, $\tilde{\tau}_{CB} = \tilde{\tau}^*_{BC} = e^{i\Phi/3}(1 + e^{i\mathbf{k}\cdot\boldsymbol{a}_2})$, $\tilde{\tau}_{AC} = \tilde{\tau}^*_{CA} = e^{i\Phi/3}(1 + e^{i\mathbf{k}\cdot\boldsymbol{a}_3})$. In the most general case, we calculate the band structure and the single-particle wavefunctions by diagonalizing the 6×6 matrix $\hat{\sigma}_z\hat{h}(\mathbf{k})$ numerically.

Analytical calculation of the band structure close to the symmetry points

One can gain much insight on the array dynamics, including the stability requirements and the array topology, by calculating analytically the band strucure at the symmetry points. This is a particularly easy task at the rotational symmetry points Γ, K, and K'. There, the hopping matrix $\hat{\tau}$ is diagonal in the basis of the quasi-angular momentum eigenstates. Thus, in this basis the Hamiltonian becomes block diagonal with 2-dimensional blocks. Each block is described by a two-mode squeezing Hamiltonian, except for the quasi-momentum $\mathbf{k} = \Gamma$ and $m = -m_\nu$, when it is a single mode squeezing Hamiltonian

$$\begin{aligned} \hat{H}_{\mathrm{L}}(\mathbf{k}) &= -\sum_m [\nu_{\mathrm{on}} + \nu'_{\mathrm{off}}(\tau_m + \tau_{m-m_\nu})]\hat{\tilde{a}}^\dagger_{\mathbf{k},m}\hat{\tilde{a}}^\dagger_{-\mathbf{k},m_\nu-m} + \mathrm{H.\,c.}\,, \quad \text{for} \quad \mathbf{k} = K, K', \\ \hat{H}_{\mathrm{L}}(\Gamma) &= -\Big\{\sum_{m\neq m_\nu} [\nu_{\mathrm{on}} + \nu'_{\mathrm{off}}(\tau_m + \tau_{m-m_\nu})]\hat{\tilde{a}}^\dagger_{\Gamma,m}\hat{\tilde{a}}^\dagger_{\Gamma,m_\nu-m} - (\nu_{\mathrm{on}}/2 + \nu'_{\mathrm{off}}\tau_{m_\nu})\hat{\tilde{a}}^{\dagger 2}_{\Gamma,-m_\nu}\Big\} + \mathrm{H.\,c.}\,. \end{aligned}$$

where $\hat{\tilde{a}}_{\mathbf{k},m}$ is the creation operator of an excitation with quasimomentum $\mathbf{k}$ ($\mathbf{k} = \Gamma, K, K'$) and quasi-angular momentum m. Moreover, τ_m indicates the corresponding eigenvalue of the hopping matrix $\hat{\tau}$: $\tau_0(\Gamma) = 4$, $\tau_1(\Gamma) = \tau_{-1}(\Gamma) = \tau_1(K) = \tau_{-1}(K') = -2$, $\tau_0(K) = \tau_1(K') = \tau_0(K') = \tau_{-1}(K) = 1$. By diagonalizing the squeezing Hamiltonian we find the general expression for the eigenvalues

$$E_m = -J\frac{\tau_m - \tau_{m-m_\nu}}{2} + \left[\left(\omega_0 - J\frac{\tau_m + \tau_{m-m_\nu}}{2}\right)^2 - |\nu_{\mathrm{on}} + \nu'_{\mathrm{off}}(\tau_m + \tau_{m-m_\nu})|^2\right]^{1/2}. \tag{3}$$

From an analogous calculation we obtain the spectrum of the effective model at the rotation symmetry points

$$\tilde{E}_m = \left[\left(\tilde{\omega} - \tilde{J}\tilde{\tau}_m(\Phi)\right)^2 - \tau_m^2\tilde{\nu}^2\right]^{1/2} \tag{4}$$

where $\tilde{\tau}_m(\mathbf{k},\Phi)$ are the eigenvalues of $\hat{\tilde{\tau}}$: $\tilde{\tau}_0(\Gamma) = 4\cos[\Phi/3]$, $\tau_{\pm 1}(\Gamma) = 4\cos[2\pi/3 \mp \Phi/3]$, $\tau_1(K) = \tau_{-1}(K') = -2\cos[\Phi/3]$, $\tau_1(K') = \tau_0(K) = 2\cos[\pi/3 - \Phi/3]$, $\tau_{-1}(K) = \tau_0(K') = 2\cos[\pi/3 + \Phi/3]$.

Stability analysis

The system is stable when all eigenvalues of $\hat{\sigma}_z\hat{h}(\mathbf{k})$ are real. If all eigenenergies of the unperturbed Hamiltonian $\hat{H}_0$ have the same sign the parametric interaction is off-resonant and the system is stable if the parametric couplings are below a finite threshold. On the contrary, if the unperturbed band touches the zero-energy axis, the parametric interaction is resonant for the zero energy modes leading to an instability for any arbitrarily small value of the coupling. Thus, the parametric instability sets an upper limit to the hopping J. For concreteness, we consider a positive onsite energy ω_0 (corresponding to a red detuned drive). In this case, all eigenenergies of $\hat{H}_0$ are positive if $J < \omega_0/4$. In this case, the system is stable for sufficiently small values of the parametric couplings $\nu_{\rm on}$ and $\nu_{\rm off}$. Nevertheless, the threshold of an instability is reached as soon as the lowest eigenenergy of a particle-type band becomes zero for a finite value of the parametric couplings $\nu_{\rm on}$ and $\nu_{\rm off}$. For the parameters of the topological phase diagram shown in Fig. 3 of the main text the lowest band touches the zero energy axis at the Γ point. Thus, we can find an analytical expression for the instability threshold using the solutions at the rotational symmetry points Eqs. (3,4). In Fig. 3(a) the state with zero energy at the border of the unstable region has zero quasimomentum and an anti-vortex. Thus, the instability threshold is given by setting $E_{-1}(\Gamma) = 0$. In Fig. 3(b) the state with zero energy has zero quasi-momentum and quasi-angular momentum. By setting $\tilde{E}_0(\Gamma) = 0$, we find a simple expression for the instability threshold, $\tilde{\nu} = \tilde{\omega}/4 - \tilde{J}\cos[\Phi/3]$.

Supplementary Note 2: Details of the definition and properties of the symplectic Chern number

Berry phase of a Bogoliubov quasi-particle

For an excitation conserving Hamiltonian, the Chern number of the m-th band can be viewed as a sum of Berry phases accumulated on a set of closed loops covering the whole Brillouin zone. In this case, the state which accumulates the relevant Berry phase is the m-th eigenstate of the single-particle Hamiltonian $\hat{h}_{\mathbf{k}}$ (a block with quasimomentum $\mathbf{k}$ of the single-particle Hamiltonian $\hat{h}$). Below, we show that one can naturally extend this definition of the Chern number to any bosonic Hamiltonian including anomalous terms by identifying the relevant Berry phase in a second-quantized setting.

For each quasi-momentum $\mathbf{k}$, the second-quantized block $\hat{H}_{\mathbf{k}} = \hat{\Psi}_{\mathbf{k}}^\dagger\hat{h}(\mathbf{k})\hat{\Psi}_{\mathbf{k}}$ of the full bosonic Hamiltonian $\hat{H}$ is a six-mode squeezing Hamiltonian. If we regard the quasi-momentum $\mathbf{k}$ as an external parameter, we can ask ourself what is the additional Berry phase accumulated by a single Bogoliubov quasi-particle in a specific band n while the quasi-momentum is varied adiabatically over a closed loop. In other words, we calculate the Berry phase accumulated by the many-body state $\hat{\beta}_{\mathbf{k},n}^\dagger|S_{\mathbf{k}}\rangle$ where $|S_{\mathbf{k}}\rangle$

is the Bogoliubov vacuum. We find

$$\begin{aligned}
\varphi_n &= i\oint\langle S_{\mathbf{k}}|\hat\beta_{\mathbf{k},n}\nabla_{\mathbf{k}}\hat\beta^\dagger_{\mathbf{k},n}|S_{\mathbf{k}}\rangle\cdot d\mathbf{k}\\
&= i\oint\langle S_{\mathbf{k}}|[\hat\beta_{\mathbf{k},n},\nabla_{\mathbf{k}}\hat\beta^\dagger_{\mathbf{k},n}]|S_{\mathbf{k}}\rangle\cdot d\mathbf{k}+i\oint\langle S_{\mathbf{k}}|\nabla_{\mathbf{k}}|S_{\mathbf{k}}\rangle\cdot d\mathbf{k}\\
&= i\oint\left(u^*_{\mathbf{k},n}[s]\nabla_{\mathbf{k}}u_{\mathbf{k},n}[s]-v^*_{\mathbf{k},n}[s]\nabla_{\mathbf{k}}v_{\mathbf{k},n}[s]\right)\cdot d\mathbf{k}+i\oint\langle S_{\mathbf{k}}|\nabla_{\mathbf{k}}|S_{\mathbf{k}}\rangle\cdot d\mathbf{k}\\
&= \oint\mathcal{A}_n\cdot d\mathbf{k}+i\oint\langle S_{\mathbf{k}}|\nabla_{\mathbf{k}}|S_{\mathbf{k}}\rangle\cdot d\mathbf{k}.
\end{aligned}$$

In the second line we have used that $\hat\beta_{\mathbf{k},n}|S_{\mathbf{k}}\rangle = 0$ (by definition of the vacuum). We note that the Bogoliubov vacuum $|S_{\mathbf{k}}\rangle$ is quasi-momentum dependend and could possibly accumulate a Berry phase by its own, $i\oint\langle S_{\mathbf{k}}|\nabla_{\mathbf{k}}|S_{\mathbf{k}}\rangle\cdot d\mathbf{k}\neq 0$. However, the Berry phase of interest is the additional Berry phase accumulated by the quasi-particle added over the Bogoliubov vacuum.

Properties of the symplectic Chern numbers

Taking into account the orthonormality condition Equation (6) of the main text, one can immediately prove that the Chern numbers have the usual properties: (i) They are integer numbers; (ii) After a phase transition where two or more bands touch the individual Chern number of the band involved in the crossings may change but their sum does not change. Since the crossing of a particle and hole band lead to an instability rather than a phase transition, the sum of the Chern numbers over the particle bands is zero.

Supplementary Note 3: Details of the calculation of the topological phase diagrams

Symmetry of the topological phase diagram under synthetic magnetic field inversion

In the topological phase diagram shown in Fig. 3(b) all Chern numbers change sign if the direction of the synthetic gauge field is inverted, $\Phi\to-\Phi$. This has a simple explanation: To change the sign of the flux Φ and of the quasimomentum $\mathbf{k}$ corresponds to taking the complex conjugate of the BdG Hamiltonian in momentum space, $\hat{\hat{h}}(-\mathbf{k},-\Phi)=\hat{\hat{h}}^*(\mathbf{k},\Phi)$, c.f. Supplementary Equation (2). It follows that the single-particle eigenfunctions for opposite values of the flux and of the quasi-momentum are also related by complex conjugation, $|\mathbf{k}_n(\Phi)\rangle\rangle = (|-\mathbf{k}_n(-\Phi)\rangle\rangle)^*$. From the definition of the Chern numbers, c. f. Equation (7) of the main text, it immediately follows that the Chern numbers change sign under inversion of the synthetic gauge field Φ.

Border of the different topological phases

At a border of a topological phase transition a pair of Chern numbers can change their values because the corresponding bands touch. Generally speaking bands tend to repel each other rather than crossing. However, at a lattice symmetry point this phenomenon does not necessarily occur because the interaction of a pair of bands can be prevented by a selection rule. In particular, at the rotational symmetry points K, K', and Γ, a hole with quasi-angular momentum m can only be converted into a particle with quasi-angular momentum $m+m_\nu$. We note that due to inversion symmetry the bands must touch simultaneously at the symmetry points K and K'. We refer to the set of parameters where the bands touch at the symmetry points K and K' (Γ) as K-lines (Γ-lines). When also time-reversal symmetry is

present there is band crossing at all rotational symmetry points. We refer to the set of parameters where time-reversal symmetry occurs as $\mathcal{T}$-lines. In addition, a pair of bands can touch at one of the three M points where one sublattice is decoupled from the remaining two sublattices (a particle or hole on that sublattice can not hop on the remaining sublattices). We note that due to rotational symmetry a pair of bands should touch simultaneously at all three M points. We refer to the set of parameters where a pair of bands touch at the M points as M-lines.

In our highly symmetric system, we expect most of the crossings to occur at a symmetry point. However, we note that accidental crossings away from any symmetry point are not forbidden. Indeed most (but not all) borders of the different topological phases in Fig. 2 can be identified with $\mathcal{T}$-lines, K-lines, Γ-lines, or M-lines as explained below.

We first focus on the effective model. The vertical lines $\Phi = 0, \pm 3\pi$ are $\mathcal{T}$-lines (there is time-reversal symmetry because the hopping amplitude $\tilde{J}$ is real). One can also easily recognize the M-lines because they are horizontal. This must be the case because the spectrum at a M-point where a sublattice decouples from the remaining sublattices does not depend on the flux Φ. Indeed, there is such a horizontal line in the phase diagram of Fig. 3(b). We note that it appears for $\tilde{\nu}^2 \approx |\tilde{J}_{ij}|\tilde{\omega}$. Below, we show that this analytical expression holds when the $\hat{\alpha}$ quasi-particles are described by an effective particle-conserving Hamiltonian. We can also find an analytical expression for the K-line and the Γ-lines as explained below.

We initially focus on the Γ-lines. We regard the band crossing condition of a pair of levels with quasi-angular momentum m and m', $\tilde{E}_m(\Gamma) = \tilde{E}_{m'}(\Gamma)$ as an implicit equation for the parametric coupling $\tilde{\nu}$ as a function of the flux Φ. We take advantage of the analytical expression for the spectrum at the rotational symmetry points Supplementary Equation (4) to solve this equation. For $m = 0$ and $m' = -1$, we find exactly one real positive solution

$$\tilde{\nu} = \frac{1}{2\sqrt{3}}\left[\left(\omega_0 - 4\tilde{J}\cos\frac{\Phi}{3}\right)^2 - \left(\omega_0 - 4\tilde{J}\cos\frac{2\pi+\Phi}{3}\right)^2\right]^{1/2}$$

in the intervals $-3\pi \leq \Phi \leq -\pi$ and $2\pi \leq \Phi \leq 3\pi$. Keeping in mind that the phase diagram is periodic with period 6π, this solution can be thought of as a single Γ-line which goes (for increasing flux) from $\tilde{\nu} = 0$ at $\Phi = 2\pi$ back to $\tilde{\nu} = 0$ at $\Phi = 5\pi$ ($\Phi = -\pi$). Indeed, such a line is visible in the phase diagram of Fig. 3(b). From the implicit equations $\tilde{E}_m(\mathbf{k}) = \tilde{E}_{m'}(\mathbf{k})$, $\mathbf{k} = \Gamma, K$ one can find similar formulas for the remaining Γ-line and the K-lines. In particular, the other Γ-line corresponds to the crossings of the levels with angular-momentum $m = 1$ and $m = 0$ and goes from $\tilde{\nu} = 0$ at $\Phi = \pi$ back to $\tilde{\nu} = 0$ at $\Phi = 4\pi$ ($\Phi = -2\pi$), see also Fig. 3(b). There is not a third Γ-line because the levels with quasi-angular momentum $m = 1$ and $m = -1$ are degenerate only on the $\mathcal{T}$-lines. Likewise, one can show that the K-lines go from $\tilde{\nu} = 0$ at $\Phi = -\pi$ back to $\tilde{\nu} = 0$ at $\Phi = \pi$ and from $\tilde{\nu} = 0$ at $\Phi = -2\pi$ back to $\tilde{\nu} = 0$ at $\Phi = 2\pi$, respectively. We note that the formulas for the band crossings are exact and valid for an arbitrary ratio of $\tilde{J}/\tilde{\omega}$. However, if $\tilde{J}/\tilde{\omega}$ is above a finite threshold the unstable region may overlap with the band crossings and not all topological phases will be present in the phase diagram.

Above we have identified all lines forming the border of the different topological phases in Fig. 3(b) except for the lines which appear above the M-lines very close to the $\mathcal{T}$-lines and surrounds the white areas of the topological phase diagram Fig. 3(b). These lines correspond to accidental crossings which occur away from any symmetry point. They enclose four different topological phases (which are not listed in our legend for brevity).

Next, we discuss the topological phase diagram of the original model. For a pump circulation $m_\nu = 1$ ($m_\nu = -1$), the resulting effective flux Φ is positive (negative), see Equation (11) of the main text. Thus, topological phase diagram of the original model is a deformed version of the right (left) half of the effective model phase diagram, see panel (a) of Fig. 2 for the case $m_\nu = 1$ ($m_\nu = -1$). The case $m_\nu = 0$ on the other hand is mapped onto the $\mathcal{T}$-lines of the effective diagram.

A remarkable feature of our model is that there is only a single topological phase for any fixed value m_ν of the pump circulation if the off-diagonal parametric terms are not present ($\nu_{\text{off}} = 0$): $\mathbf{C} = (\mp 1, 0, \pm 1)$ for $m_\nu = \pm 1$ and $\mathbf{C} = (0, 0, 0)$ for $m_\nu = 0$. This is reminiscent of the anomalous Quantum Hall effect on

a Kagome lattice with nearest neighbor hoppings (OMN model) where the topological phase is uniquely determined by the sign of the magnetic flux piercing a triangular plaquette, $\mathbf{C} = (\mp 1, 0, \pm 1)$ if the flux is positive or negative, respectively. Indeed, for small squeezing $\nu_{\rm on}, J \ll \omega_0$, the parametric interaction effectively induces a small synthetic gauge field with a positive flux for $m_\nu = 1$. This can be easily seen by switching to the effective description and neglecting the residual parametric terms. For concreteness we consider the case $m_\nu = 1$. For small squeezing and $\nu_{\rm off} = 0$ we are somewhere close to $\tilde{\nu} = 0$, $\Phi = 2\pi$ inside the topological phase $\mathbf{C} = (-1, 0, 1)$ at the bottom right corner of the effective diagram. From the above analysis of the effective diagram we know that a topological phase transition can occur only if we cross a Γ or $\mathcal{T}$-line. However, from the analytical solution of the spectrum at the rotational symmetry points of the original model Supplementary Equation (3) we see that such crossings never occur on the $\nu_{\rm off} = 0$ axis. Thus, there in no topological phase transition even for large squeezing if $\nu_{\rm off} = 0$.

Effective excitation-conserving Hamiltonian

When $\tilde{\omega}$ is much larger than $|\tilde{J}|$ and $\tilde{\nu}$ one can derive an effective excitation conserving Hamiltonian. In the regime where $\tilde{\nu} \gg |\tilde{J}|$ it is not enough to keep the excitation conserving terms in Equation (8) of the main text but one should also include the leading order correction in $\tilde{\nu}/\tilde{\omega}$. We arrive at the excitation conserving Hamiltonian with next-nearest-neighbor hoppings,

$$\hat{H}_{\rm RW} = \sum_{\mathbf{j}} \bar{\omega} \hat{\alpha}^\dagger_{\mathbf{j}} \hat{\alpha}_{\mathbf{j}} - \sum_{\langle \mathbf{j},\mathbf{l} \rangle} \bar{J}^{(1)}_{\mathbf{jl}} \hat{\alpha}^\dagger_{\mathbf{j}} \hat{\alpha}_{\mathbf{l}} - \sum_{\langle\langle \mathbf{j},\mathbf{l} \rangle\rangle} \bar{J}^{(2)}_{\mathbf{jl}} \hat{\alpha}^\dagger_{\mathbf{j}} \hat{\alpha}_{\mathbf{l}}. \quad (5)$$

Here, $\langle\langle \mathbf{j}, \mathbf{l} \rangle\rangle$ indicates the sum over next-nearest-neighbor sites and

$$\begin{aligned} \bar{\omega} &= \tilde{\omega} - \frac{2\tilde{\nu}^2}{\tilde{\omega}}, \qquad \bar{J}^{(2)}_{\mathbf{jl}} = \frac{\tilde{\nu}^2}{2\tilde{\omega}} \\ \bar{J}^{(1)}_{\mathbf{jl}} &= \tilde{J}_{\mathbf{jl}} + \frac{\tilde{\nu}^2}{2\tilde{\omega}}. \end{aligned}$$

In this simplified picture it is straightforward to calculate the band structure at the M points and finding the bad degeneracy condition $\tilde{\nu}^2 = |\tilde{J}_{\mathbf{jl}}|\tilde{\omega}$ which leads to the horizontal line in the topological phase diagram of the effective model.

Supplementary Note 4: Bulk-boundary correspondence

It is well known that in a system with a boundary, the net number of edge states (the number of right-movers minus the number of left-movers) in a bulk band gap is a topological invariant [1]. This statement is based on the sole assumption that the band structure and the corresponding eigenvectors change smoothly in the presence of a local perturbation that does not close a gap. Thus, it clearly applies to any quadratic Hamiltonian. For the special case of an excitation-conserving insulator or a superconductor the bulk boundary correspondence expresses such topological invariant in terms of the Chern numbers: the net number of edge states in a band gap coincides with the sum of the Chern numbers of all bands below that band gap. Here, we explicitly show that the bulk-boundary correspondence is still valid for our model where anomalous terms are present.

We start noticing that the wavefunctions of the RWA Hamiltonian Supplementary Equation (5) depend only on two parameters: the phase $\Phi/3$ of $\tilde{J}$ and the dimensionless next-nearest-neighbor coupling $\tilde{\nu}^2/(|\tilde{J}|\tilde{\omega})$. By calculating the phase diagram as a function of these parameters [not shown] we see that it supports all topological phases present in the topological phase diagram of the effective Hamiltonian for the $\hat{\alpha}$-quasiparticles [our full model without approximations]. Thus, we can continously interpolate between the two Hamiltonians without crossing any topological phase transition [by sending $\tilde{\omega} \to \infty$ while also tuning Φ and $\tilde{\nu}^2/(\tilde{\omega}|\tilde{J}|)$ to stay in the same topological phase]. Keeping in mind that the bulk-boundary correspondence holds for the excitation-conserving Hamiltonian Supplementary Equation

(5) and that the net number of edge states does not change during the interpolation [unless a gap is closed], we can conclude that such correspondence is valid for our model even for small $\tilde{\omega}$ where the RWA leading to Supplementary Equation (5) is not a good approximation.

We note that the above reasoning combined with the assumption that a continuous interpolation between any quadratic bosonic Hamiltonian and an excitation conserving Hamiltonian is always possible without closing any band gap, leads to the general validity of the bulk-edge correspondence.

Supplementary References

[1] Hasan M. Z. & Kane C. L., Colloquium: Topological insulators. *Rev. Mod. Phys.* **82**, 3045 (2010).
